# Tuning the Mechanical Properties of a DNA Hydrogel in Three Phases Based on ATP Aptamer

**DOI:** 10.3390/ijms19061633

**Published:** 2018-05-31

**Authors:** Hengyuan Liu, Tianyang Cao, Yun Xu, Yuanchen Dong, Dongsheng Liu

**Affiliations:** 1 Department of Chemistry, Tsinghua University, Beijing 100084, China; hy-liu14@mails.tsinghua.edu.cn (H.L.); caoty15@mails.tsinghua.edu.cn (T.C.); dongyuanchen@outlook.com (Y.D.); 2 Center for Medical Device Evaluation, China Food and Drug Administration, Building 1, Qixiang Road, Beijing 100084, China; xuyun@cmde.org.cn

**Keywords:** nucleic acid nanotechnology, DNA nanotechnology, DNA hydrogel

## Abstract

By integrating ATP aptamer into the linker DNA, a novel DNA hydrogel was designed, with mechanical properties that could be tuned into three phases. Based on the unique interaction between ATP and its aptamer, the mechanical strength of the hydrogel increased from 204 Pa to 380 Pa after adding ATP. Furthermore, with the addition of the complementary sequence to the ATP aptamer, the mechanical strength could be increased to 570 Pa.

## 1. Introduction

Hydrogels are three-dimensional polymeric networks containing a significant amount of water [1,2]. The natural extracellular matrix (ECM) is well known, due to its fundamental biological properties, which provide essential support to the cells in living organs, facilitating the mechanical maintenance, signal, nutrition, oxygen, and waste transportation [3,4,5,6]. Many synthetic hydrogels have been designed to mimic ECM and applied in cell culture [7,8,9,10], diagnostics [11,12,13], and drug delivery [14,15,16,17]. The mechanical properties of hydrogel play critical roles in modulating cell–matrix interactions, as well as regulating biological processes, including cell proliferation, spread, and differentiation [18,19,20]. Therefore, constructing hydrogels with similar strengths as an ECM, with regard to physical condition, is an important target.

During the last few decades, DNA has been developed as an essential building block for fabricating delicate devices and materials [21,22,23,24,25]. Recently, novel DNA-based hydrogels have been reported with many unique properties [26,27,28], including rapid formation [29,30], tunable mechanical strength [31], thixotropy, and good permeability to macromolecules. Due to the functionality and programmability of DNA, innovative functional hydrogels were also prepared. For example, Li et al. reported a writable polypeptide–DNA hydrogel by designing multi-modification sites in 2015 [32]. In addition, Zhou et al. then designed a pH-responsive DNA hydrogel by integrating a DNA motor based on an i-motif sequence in 2016 [33]. These hydrogels were designed with comparable mechanical properties as an ECM in their physical condition, but to tune the strength to multiple phases, in situ, has not yet been realized. Adenosine triphosphate (ATP) is a crucial biological molecule in energy transportation for metabolism [34] and cell communication [35], which can be potentially employed to responsive hydrogel systems for mechanical property tuning. The ATP aptamer is a short single DNA sequence [36], which can specifically bind ATP to form a stable framework composed of two stacked G-quartets, resulting in conformational transitions in DNA structures [37,38,39,40]. Herein, by integrating ATP aptamer into the network, an ATP-responsive DNA hydrogel was designed with three mechanical phases adjusted in situ. This work provides a general strategy for tuning the strength of DNA hydrogel in situ.

**Scheme 1:**
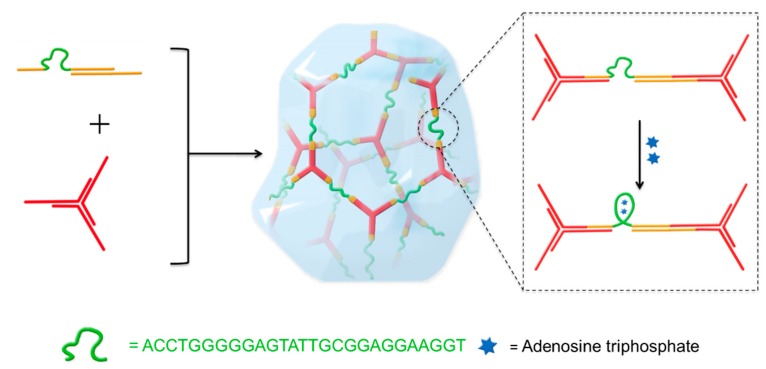
The Y-scaffold (**red line**) and linear linker (**yellow line**) were designed to form a DNA hydrogel by hybridization of the reactive ends. The ATP aptamer sequence (**green line**) was inserted into the region of the linker. On addition of adenosine triphosphate (**blue star**), the conformational transition of the aptamer had a great influence on the mechanical properties of the DNA hydrogel.

The strategy to integrate an ATP aptamer sequence into the backbone of hydrogel network is illustrated in Scheme 1. A previously reported DNA hydrogel with high permeability to small molecules and macromolecules up to 20 kDa [7] is chosen as a basic hydrogel system, which is composed by five different DNA strands: B1, B2, Y1, Y2, and Y3. A rigid Y-scaffold structure (red) was assembled from Y1, Y2, and Y3, leaving a 12 nt single-stranded sticky end at the end of each arm. B1 and B2 could form a double helix linker (yellow) with another 12 nt single strand complementary to the Y-scaffold sticky ends, which can further crosslink into the hydrogel. In the linker, a 27 base ATP aptamer sequence (green, detailed information in Table S1) is inserted into the B1 sequence, and thus, to fix the aptamer sequence into the backbone of the hydrogel in a flexible single strand state with no ATP binding. Due to the high permeability of the hydrogel system, ATP can easily diffuse through the network after addition and bind to the aptamer, leading to the aptamer transition from single strand to a more rigid secondary structure. Furthermore, the conformational change of the aptamer sequence would shorten the distance between the crosslinking points in the hydrogel network. Therefore, the synergistic effect of the aptamer conformational transition within the whole network will improve the macroscopic mechanical properties of the hydrogel to a second phase. In this way, DNA hydrogels could be fabricated with tuneable mechanical properties in situ, and a fast ATP-responsiveness.

## 2. Results and Discussion

**Figure 1:**
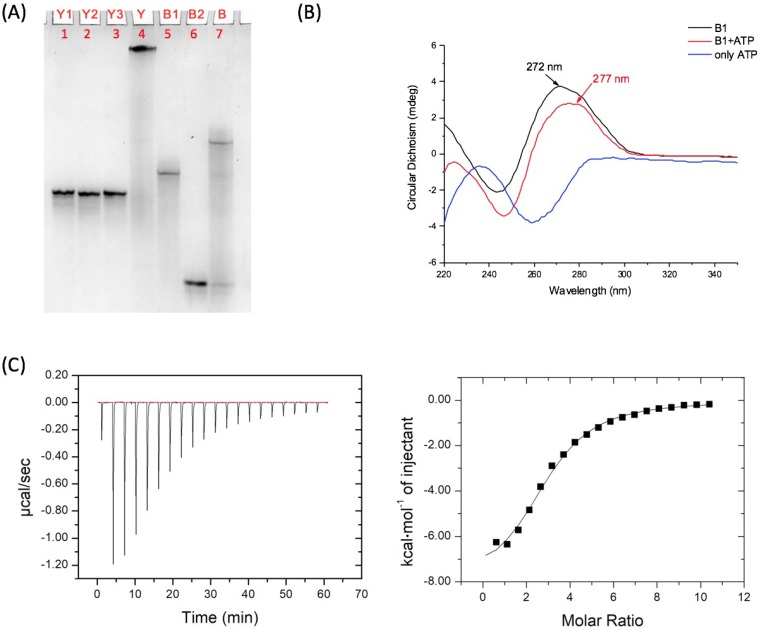
Characterization of the DNA assemblies and ATP aptamer structure. (**A**) Characterization of Y-scaffold and B-linker by 20% native PAGE. Three ssDNAs Y1 (**Lane 1**), Y2 (**Lane 2**), and Y3 (**Lane 3**) hybridized to form the Y-scaffold (**Lane 4**), and two ssDNAs B1 (**Lane 5**) and B2 (**Lane 6**) assembled into the linear B-linker (**Lane 7**); (**B**) CD spectra to characterize the conformation of the aptamer sequence in DNA strand B1 with or without ATP; and (**C**) Isothermal titration calorimetry (ITC) data (**left**) and fitting curve (**right**) of binding strength between B1 and ATP.

In a typical experiment, the ssDNAs (single-stranded DNAs) Y1, Y2, and Y3 were synthesized, purified, and then mixed stoichiometrically in Tris-HCl buffer (pH = 8.3, 300 mM NaCl and 5 mM MgCl_2_) with a final concentration of 60 μM for each strand. The mixture was heated to 95 °C for 5 min, and subsequently cooled to room temperature, slowly, to form the Y-scaffold. Following the same procedure, the linker was formed by mixing B1 and B2 stoichiometrically. Polyacrylamide gel electrophoresis (PAGE) was applied to verify the assembly. As shown in Figure 1A, the clean bands in Lanes 1–3, 5, and 6 indicate the successful synthesis and purification of five single strands. The Y-scaffold (Lane 4) and the linker (Lane 7) migrated slower than the single strands, which demonstrated that all DNA assembled-structures were formed, as designed, in high yield.

In addition, circular dichroism (CD) was also used to characterize the binding process. When the binding between ATP and aptamer leads to a conformational change, the CD signal will be different. A specific protocol was designed for performing the CD measurements. Single strand B1 (5 μM) and linker (B1/B2 assembly, 5 μM) were incubated with 1 mM ATP for 30 min at room temperature, respectively, in 20 mM Tris-HCl buffer with 5 mM MgCl_2_ and 300 mM NaCl. The samples were then measured by CD using a 1 millimeter optical path quartz cell in a wavelength range from 220 nm to 350 nm, with a scanning rate of 60 nm per min. As shown in Figure 1B, for the pure B1 DNA strand, there was a positive peak at 272 nm in the spectrum. With the addition of ATP, the peak shifted to 277 nm. For the linker (B1/B2 assembly), a similar shift has been observed from 273 nm to 279 nm (as shown in Figure S1). We also tested the CD signals of a random DNA sequence with and without ATP as a control. The results are shown in Figure S2, and a certain shift has also been observed (277 nm to 280 nm). This can be explained by the fact that the CD signals are sequence dependent, and the existence of ATP has also affected the adsorption, which decreases the change in CD signal from aptamer conformational change.

To fully prove the binding between ATP and the aptamer sequence, we applied isothermal titration calorimetry (ITC) to determine the binding constant. ATP was titrated into DNA (aptamer sequence) in the 20 mM Tris-HCl buffer (pH = 8.3, with 5 mM MgCl_2_, 300 mM NaCl). The titration curve was shown as Figure 1C, and the measured binding constant is approximately 8.97 × 10^4^ M^−1^, which is close to the previous reported data [36].

**Figure 2:**
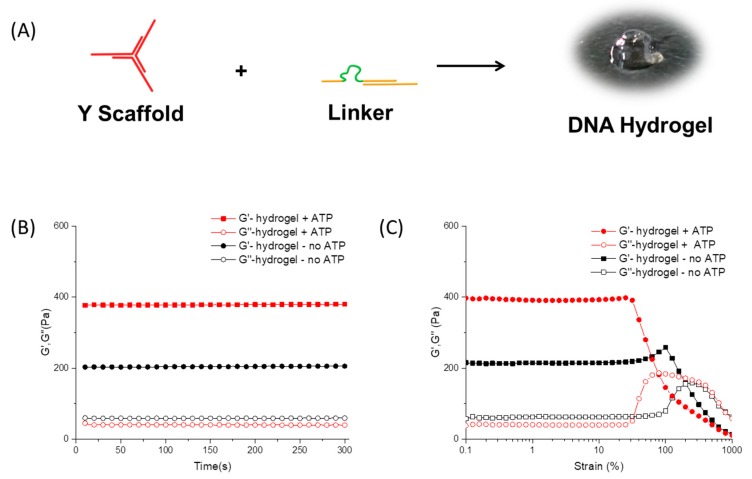
Fabrication and characterization of DNA hydrogel. (**A**) Formation of hydrogel by mixing the Y-scaffold (500 μM) and B-linker (750 μM) in 20 mM Tris-HCl buffer; (**B**) The mechanical strength of the hydrogels with or without ATP was characterized by a time sweep test with a fixed strain of 1% and frequency of 1 Hz at 25 °C; and (**C**) The shear-thinning property of the hydrogels with or without ATP was measured by a strain sweep test from 0.1% to 1000% with a fixed frequency of 1 Hz at 25 °C.

The DNA hydrogels were prepared following a simple mixing protocol. Three single-stranded DNA, Y1, Y2, and Y3, were mixed in a stoichiometric ratio, and then frozen dry to a white solid. After being redissolved in 20 mM Tris-HCl buffer (pH = 8.3, with 5 mM MgCl_2_, 300 mM NaCl), the sample was heated to 95 °C for 5 min followed by cooling to room temperature to form Y-scaffold. The B-linker solution was also prepared in a similar manner. By simply mixing the Y-scaffolds and B-linker solutions together and vibrating for 1 min, the solution turned into a transparent gel, as shown in Figure 2A. In the typical hydrogel, the final concentration of Y-scaffold and B-linker were 500 μM and 750 μM, respectively.

To tune the mechanical strength of the hydrogel in situ, 1 μL 500 mM ATP solution in Tris-HCl buffer was added to 39 μL of the prepared DNA hydrogel at room temperature, and incubated for 30 min. Tris-HCl buffer (1 μL) was separately added to another 39 μL hydrogel as a control. Oscillatory shear test with a rotational rheometer was applied to measure the mechanical strength of the hydrogels. The time sweep test was carried out at a fixed strain of 1% and frequency of 1 Hz at 25 °C, with a 150 μM gap between geometries. As shown in Figure 2B, the storage modulus (G’) of both samples is much higher than the loss modulus (G’’), confirming the hydrogel formation in both conditions. The G’ and G’’ of the original pure DNA hydrogel without ATP are 204 Pa and 59 Pa, respectively. By contrast, for the hydrogel with ATP, the G’ is higher (380 Pa), and the G’’ is lower (37 Pa). The difference of mechanical strength can be explained by the conformational change of the DNA secondary structure. In the control group, the network of pure DNA hydrogel is more open and flexible, due to the existence of the single strand ATP aptamer sequence. More energy can be dissipated by the single strand structure, thus, the resultant hydrogel has a relatively higher G”. With the addition of ATP, the aptamer sequence formed a more rigid secondary structure, and as a result, the hydrogel exhibits enhancement of G’.

The mechanical properties were also studied with a strain sweep test at a fixed frequency of 1 Hz. As shown in Figure 2C, the value of G’ remains higher than G’’ for both samples at low strain, indicating that the hydrogel is stable under small deformation. However, when the strain increases, the G’ decreases and the G’’ increases. When the deformation exceeds a critical value, the two curves of G’ and G’’ intersect, and finally G’’ becomes higher than G’, which indicates that the samples turned from a gel state into a solution state. For the pure DNA hydrogel without ATP, the gel-to-sol transition occurred at a strain of 159%. This shear-thinning property could be explained by the dynamic supramolecular interactions between DNA strands. Under a large deformation, the double helix dissociates to single strands, and the network collapses, turning the hydrogel into the liquid state. The transition point of strain for the hydrogel with 12.5 mM ATP was 79%. The difference of transition point of these two hydrogels indicates that the pure DNA hydrogel without ATP was relatively tough and ductile. After the addition of ATP, the hydrogel became stronger, but more brittle.

**Figure 3:**
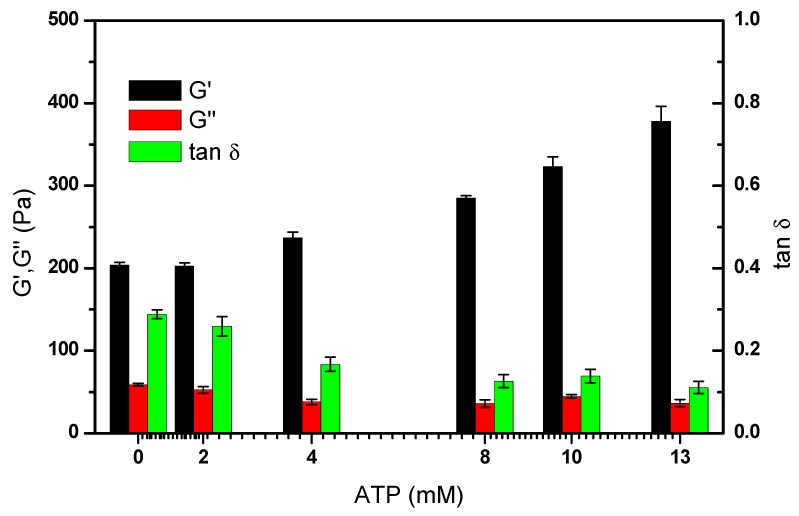
Variation tendency of the mechanical properties of DNA hydrogels, i.e., storage modulus (G’), loss modulus (G’’), and loss tangent (tan δ), under an ATP concentration gradient from 0 to 12.5 mM. All the measurements were carried out three times to get the average and statistical analysis was applied to get the error bar.

Furthermore, the ATP concentration dependent mechanical properties of the DNA hydrogel were also tested. As shown in Figure 3, in the low-level range of ATP concentrations, from 0 to 1.5 mM, G’ of the hydrogel does not change significantly, while G’’ declines with the increase in ATP concentration. It is possible that at low concentrations, only a small proportion of DNA interacts with ATP, which cannot lead to an obvious macroscopic storage modulus change. However, for those DNA strands which changed from flexible to rigid structure, the storage loss might decrease. When the concentration of ATP reaches a higher level, an obvious increase of storage modulus was observed, as shown in Figure 3, while G” and tan δ show downward trends.

**Figure 4:**
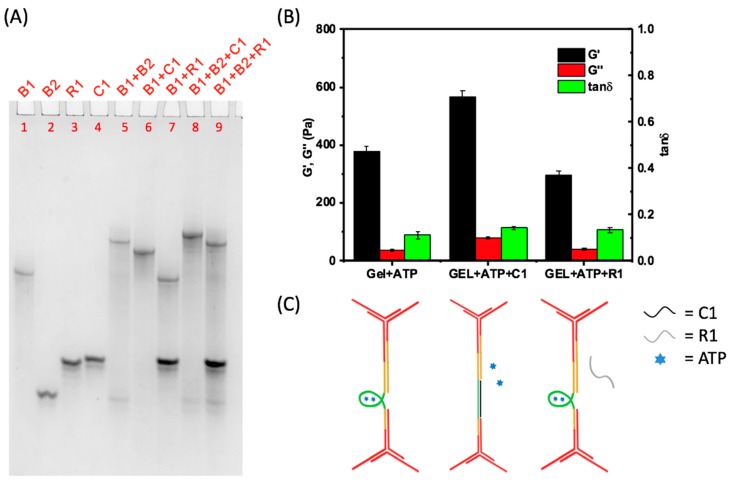
Further tuning the mechanical strength of DNA hydrogels by adding an ssDNA complementary to the aptamer sequence. (**A**) Characterization of C1B1 structure by 20% native PAGE. Four bands of ssDNAs B1 (**Lane 1**), B2 (**Lane 2**), R3 (**Lane 3**), and C1 (**Lane 4**) are clear. C1 can form structure with B1 with (**Lane 6**) or without B2 **(Lane 8**), while R1 cannot hybridize with B1 (**Lane 7** and **Lane 9**); (**B**) Study of mechanical properties of the DNA hydrogel on adding ssDNA C1 and R1. All the measurements were carried out three times to get the average and statistical analysis was applied to get the error bar; and (**C**) Illustration of microscopic conformation transition of the aptamer sequence on adding ssDNA C1 and R1.

The aptamer can not only bind ATP molecules, but also can hybridize with its complementary DNA strand. A 27 nt sequence C1 was synthesized, which is fully complementary to the aptamer sequence. As shown in Figure 4A, the clean bands in the PAGE result demonstrate that the C1 strand and the B1 strand could completely hybridize in the addition of ATP. When the complementary strand binds to the aptamer sequence, the mechanical properties of the aptamer-based hydrogel should be able to be further tuned with the C1 strand to the third phase.

To demonstrate this additional tuning, a hydrogel with ATP was prepared, and then the C1 strand was added, which leads to the third phase, with a final volume of 40 μL (Figure 4C). The ratio of C1 and B1 is 1:1. As shown in Figure 4B, the G’ of the hydrogel is 570 Pa, which is much higher than the G’ of the hydrogel with only ATP. The G’’ also increases from 37 Pa to 80 Pa with the addition of the C1 strand. To exclude the influence of mass content increase on hydrogel strength, we also designed a 27 bases strand R1 of random sequence as a control. The G’ and G’’ of the hydrogel remained about the same after adding R1. The experiment above confirms that the addition of C1 could turn the former structure into a more rigid double helix, and further enhance the mechanical strength of the hydrogel.

## 3. Materials and Methods

### 3.1. Materials

All oligonucleotides (see Table S1) were synthesized with MerMade-12 DNA synthesizer (Bioautomation, Irving, TX, USA) using a standard phosphoramidite DNA synthesis protocol and purified by HPLC (High Performance Liquid Chromatography) using water/acetonitrile/TEAA (triethylamine acetate buffer, 100 mM, pH = 7.0) as eluent. Water used in all experiments was Millipore Milli-Q deionized (18.2 MΩ·cm^−1^). Adenosine triphosphate (ATP) was purchased from Adamas. Thymidine triphosphate (TTP) was purchased from Sigma-Aldrich (St. Louis, MO, USA). All other chemical reagents were purchased from Sigma-Aldrich or Alfa Aesar (Ward Hill, MA, USA), and used as received.

### 3.2. Preparation and Characterization of DNA Assemblies

For preparation of Y-scaffold, three oligonucleotide strands, Y1, Y2, and Y3 were mixed stoichiometrically in 20 mM Tris-HCl (pH = 8.3, 5 mM MgCl_2_, 300 mM NaCl) to give a final concentration of 50 μM for each strand. Then, the mixture was heated to 95 °C and slowly cooled to room temperature. Similarly, the linker was formed by mixing of B1 and B2 stoichiometrically following the same annealing procedure. All DNA assemblies were characterized by native polyacrylamide gel electrophoresis (20%, Acrylamide/Bis-acrylamide = 19:1) in 1 × TBE(Tris-borate-EDTA) buffer with 200 V for 10 h at 4 °C. Melting points of Y-scaffold and B-linker were measured from 4 °C to 95°C with Cary100 UV–Vis spectrometer (Agilent Technologies, Palo Alto, CA, USA). The results can be found in Figure S3.

### 3.3. DNA Hydrogel Preparation

To form a 4 wt % hydrogel (40 μL), three ssDNAs, Y1, Y2, and Y3 (500 μM, 40 μL), were mixed at an equal molar ratio, and the solution was lyophilized to obtain a white solid. After adding 20 μL Tris-HCl (20 mM Tris-HCl, pH = 8.3, 5 mM MgCl_2_, 300 mM NaCl), the Y-scaffold solution (1 mM, 20 μL) was prepared, as well. The linker solution (1.58 mM, 19 μL) was prepared by mixing of B1 and B2 (750 μM, 40 μL) stoichiometrically, following a similar protocol. Then, the two solutions were mixed at room temperature with a vortex, and the mixture changed from a liquid state to a transparent gel state within several seconds. Then, 1 μL of buffer or ATP solution was added into the gel and mixed. In the control group, 1 μL of TTP solution was added in to the gel and mixed. The results can be found in Figure S4.

### 3.4. Rheological Characterization

A Kinexus Pro^+^ rheometer (Malven Instruments, Worcestershire, UK) was used to characterize the mechanical properties of the formed hydrogels. Three types of rheological experiments were performed in 8 mm parallel-plate geometry using 40 μL hydrogels (with gap size of 0.15 mm): (i) Time sweep test was carried out at a fixed strain of 1% and frequency of 1 Hz at 25 °C for 5 min; (ii) Strain sweep test was carried out from 0.1% to 1000% with a fixed frequency of 1 Hz at 25 °C.

### 3.5. Tuning Mechanical Properties of Hydrogel with ssDNA C1

We firstly prepared 30 μL hydrogel with ATP, and then add 10 μL C1 solution or buffer to get a final volume of 30 μL, and final concentration of Y of 500 mM, and B of 750 mM. The ratio of C1 to B1 is 1:1. Then, the samples were characterized by rheological tests, as described above.

## 4. Conclusions

In conclusion, an ATP aptamer sequence was integrated into a DNA hydrogel network, which resulted in a DNA hydrogel with in situ tunable mechanical properties in three phases. By adding ATP, the single-stranded aptamer sequence in the linker binds ATP and undergoes a conformational transition, leading to an enhanced storage modulus of the hydrogel from 204 Pa to 380 Pa. The mechanical properties of the hydrogel could be further improved to 570 Pa by adding a complementary sequence. It should be noted that in normal soft tissues, the strength of the ECM is usually 100–1000 Pa, which suggests this material may provide a new tool in tissue engineering. Furthermore, mechanical strength plays an important role in stem cell differentiation, so the in situ tunable property of this hydrogel at physical conditions also shows great potential in investigating this process. Additionally, this strategy could be used to integrate any other aptamer sequences, so it is a universal tool to build DNA hydrogels responsive to different molecules for potential applications.

## Supplementary Materials

Supplementary materials can be found at http://www.mdpi.com/1422-0067/19/6/1633/s1.
