# Supplementary material for: Tuning the Mechanical Properties of a DNA Hydrogel in Three Phases Based on ATP Aptamer
Source: Int J Mol Sci. 2018 May 31;19(6):1633. doi: 10.3390/ijms19061633 (PMC6032235; doi:10.3390/ijms19061633)
Supplement: Supplementary file 1 [file ijms-19-01633-s001.pdf]

International Journal of
Molecular Sciences

Supplementary Materials: Tuning the Mechanical Properties of a DNA Hydrogel in Three Phases Based on ATP Aptamer

Hengyuan Liu [1], Tianyang Cao [1], Yun Xu [2], Yuanchen Dong [1] and Dongsheng Liu [1,*]

[1] Department of Chemistry, Tsinghua University, Beijing, 100084, China; liudongsheng@tsinghua.edu.cn
[2] Center for Medical Device Evaluation, CFDA, Building 1, Qixiang Road, Beijing, 100084, China; xuyun@cmde.org.cn
* Correspondence: liudongsheng@tsinghua.edu.cn

Table S1. ssDNA sequences for preparation of DNA hydrogel.

Sample	Sequence
**Y1**	5′-CGATTGACTCTCCACGCTGTCCTAACCATGACCGTCGAAG-3′
**Y2**	5′-CGATTGACTCTCCTTCGACGGTCATGTACTAGATCAGAGG-3′
**Y3**	5′-CGATTGACTCTCCCTCTGATCTAGTAGTTAGGACAGCGTG-3′
**B1**	5′-GAGAGTCAATCGACCTGGGGGAGTATTGCGGAGGAAGGTACGCATTCGCTATA-3′
**B2**	5′-GAGAGTCAATCGTATAGCGAATGCGT-3′
**C1**	5′-ACCTTCCTCCGCAATACTCCCCCAGGT-3′
**R1**	5′-GCTAACTCTCACCACGTCTCACTCGCC-3′
**Aptamer**	5′-ACCTGGGGGAGTATTGCGGAGGAAGGT-3′
**L1**	5′-GAGAGTCAATCGTCTATTCGCATGAGAATTCCATTCACCGTAAG-3′

All oligonucleotides (Table S1) were synthesized with BioAutomation MerMade-12 DNA synthesizer using a standard phosphoramidite DNA synthesis protocol, and purified by HPLC using water/acetonitrile/TEAA (triethylamine acetate buffer, 100 mM, pH = 7.0) as eluent.

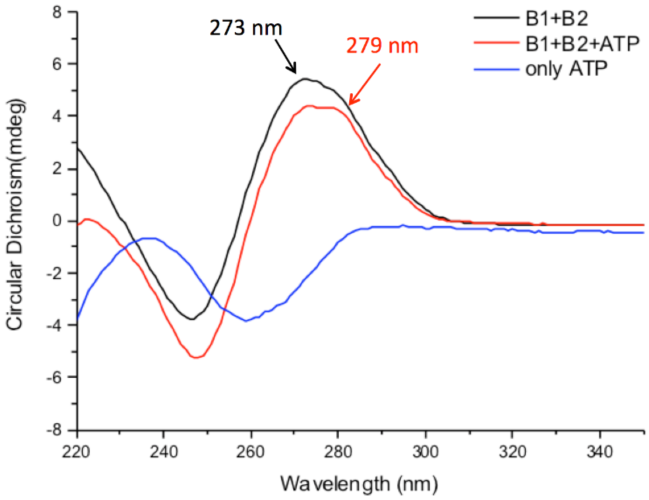

Figure S1. CD spectra of DNA assembly B1+B2, with or without ATP. B-linker (B1+B2 assembly, 5μM) were incubated with 1 mM ATP for 30 minutes at room temperature in 20 mM Tris-HCl Buffer, with 5mM $MgCl_2$ and 300 mM NaCl. The samples were then measured by CD using a 1 millimeter optical path quartz cell in a wavelength range of 220 nm to 350 nm with a scanning rate of 60 nm per minute. As shown in Figure S1, with the addition of ATP, the peak shifted from 273 to 279 nm.

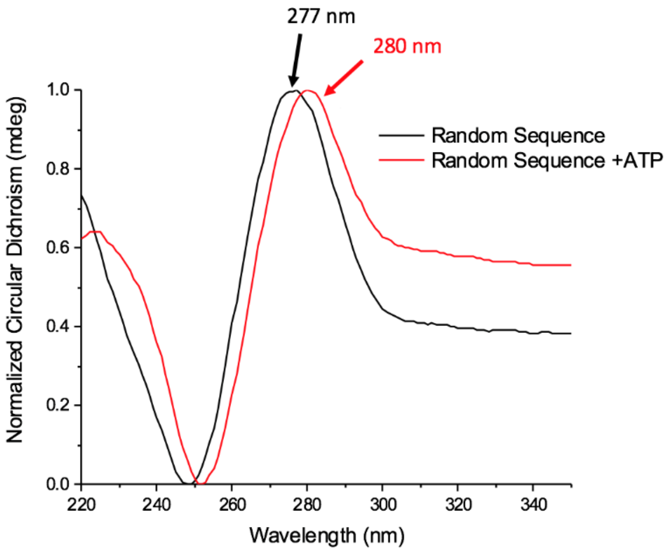

Figure S2. CD spectra of random DNA sequence with or without ATP. A random DNA sequence L1(Table S1, 5μM)was incubated with 1 mM ATP for 30 minutes at room temperature in 20 mM Tris-HCl Buffer, with 5mM $MgCl_2$ and 300 mM NaCl. The samples were then measured by CD using a 1 millimeter optical path quartz cell in a wavelength range of 220 nm to 350 nm with a scanning rate of 60 nm per minute. As shown in Figure S2, with the addition of ATP, the peak shifted from 277 nm to 280 nm. The results show that the existence of ATP can affect the CD signal, even without specific binding to DNA. Therefore, only a shift of CD signal is not enough to prove the conformational change of aptamer after binding ATP, which requires further measurements, including ITC.

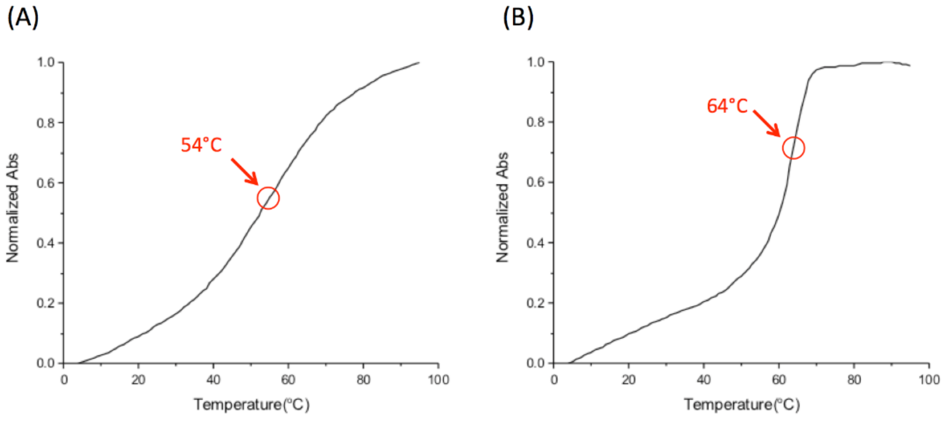

Figure S3. The melting temperatures of DNA assemblies. **(A)**The melting temperatures of B linker. **(B)** The melting temperatures of Y scaffold. Temperature ramp tests of B-linker and Y-scaffold were carried out on a Cary100 UV–Vis spectrometer (Agilent Technologies) equipped with a temperature controller, from 4 °C to 95 °C at a rate of 1 ℃/min. The melting temperature of B-linker and Y-scaffold are 54°C and 64°C, respectively.

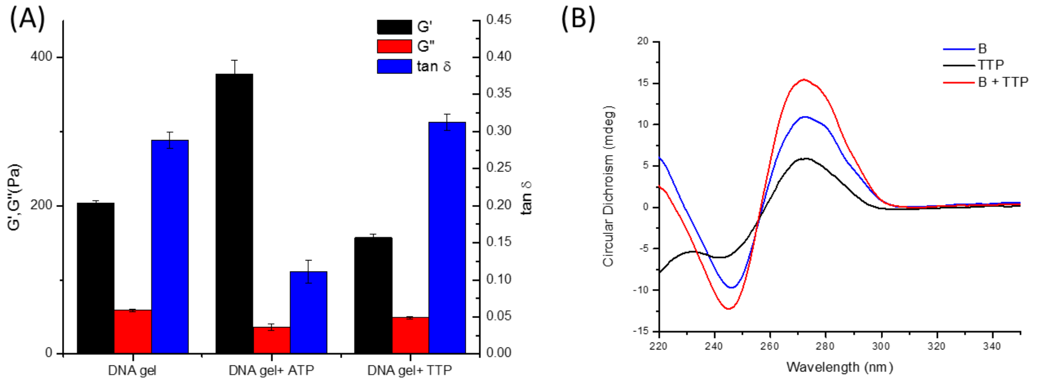

Figure S4. The change of mechanical properties and circular dichroism signal after adding TTP. **(A)** Study of mechanical properties of the DNA hydrogel after adding TTP. **(B)** CD spectra after adding TTP. As a control, 1 μL 12.5 mM thymidine triphosphate (TTP) was added to 39 μL DNA hydrogel and mixed. The rheological test at 1% strain and 1 Hz shows that after adding TTP, G′ of the DNA hydrogel slightly decreased. Circular dichroism signals of B-linker, TTP, and their mixture, were also measured from 220 nm to 350 nm. The concentration of TTP and B-linker were 1 mM and 5 μM, respectively. The samples were incubated in 20 mM Tris-HCl Buffer (pH = 8.3, with 5mM $MgCl_2$, 300 mM NaCl) at room temperature for 30 minutes before measurement. The samples were then measured by CD using a 1 millimeter optical path quartz cell in a wavelength range of 220 nm to 350 nm with a scanning rate of 60 nm per minute. The results show there is no obvious peak shift after adding TTP.
